# Designing a multiple dependent state sampling plan based on the coefficient of variation

**DOI:** 10.1186/s40064-016-3087-3

**Published:** 2016-08-30

**Authors:** Aijun Yan, Sanyang Liu, Xiaojuan Dong

**Affiliations:** School of Mathematics and Statistics, Xidian University, Xi’an, 710071 Shanxi People’s Republic of China

**Keywords:** Multiple dependent state (MDS) sampling plan, Normal distribution, Coefficient of variation (CV), Operating characteristics (OC) curve

## Abstract

A multiple dependent state (MDS) sampling plan is developed based on the coefficient of variation of the quality characteristic which follows a normal distribution with unknown mean and variance. The optimal plan parameters of the proposed plan are solved by a nonlinear optimization model, which satisfies the given producer’s risk and consumer’s risk at the same time and minimizes the sample size required for inspection. The advantages of the proposed MDS sampling plan over the existing single sampling plan are discussed. Finally an example is given to illustrate the proposed plan.

## Background

Nowadays, quality is one of the most important consumer decision factors. It has become one of the main strategies to increase the productivity of industries and service organizations. Therefore, the companies are trying to enhance the quality of their products by using various statistical techniques and tools. Acceptance sampling plans are important tools that have been widely used for lot sentencing in the industries. The inspection of the final product is always done on the basis of acceptance sampling scheme. There are two major types of acceptance sampling plans: attribute sampling plans and variable sampling plans. The major advantage of a variable sampling plan is that it has the same protection as an attribute acceptance sampling plan with a smaller sample size. When destructive testing is employed, variables sampling is particularly useful in reducing the costs of inspection. For more detail about the applications of the acceptance sampling plan can be found in Wu (2012), Liu et al. (2014), Kurniati et al. (2015), Yen and Chang (2009), and Sheu et al. (2014).

The coefficient of variation (CV), which is defined as the ratio of the standard deviation to the mean, is widely used to measure the relative variation of a variable to its mean. CV has been widely used in many practical applications. It is used as a measure of the reliability of an assay in chemistry and medicine (Reed et al. 2002), to quantify the riskiness of stocks in finance (Miller and Karson 1977), in clinical trials to account for baseline variability of measurements (Pereira et al. 2004), in physical therapy to determine sincerity of effort (Robinson et al. 1997), in quality control to seek production processes with minimal dispersion (Box 1988). Recently, Parsons et al. (2009) concluded that it was important to use CVs to assess the quality of metabonomics datasets. Kang et al. (2007) developed a Shewhart-type control chart for monitoring the CV using rational subgroups and showed the CV to be a very attractive tool in quality control.

In the literature, either the mean or the standard deviation (SD) of the quality characteristics are usually considered to measure the quality of products. However, in certain scenarios, the practitioner is not interested in the changes in the mean or the standard deviation but is instead interested in the relative variability compared with the mean (see for Yeong et al. 2015). This relative variability is called the CV. Verrill and Johnson (2007) have pointed that building materials are often evaluated not only on the basis of mean strength but also on relative variability, but laboratory techniques are often compared on the basis of their CVs. In many laboratories, the variability of the chemical assay that produces continuous-type values is summarized not by the SD but by the CV, because the SDs of such assays generally increase or decrease proportionally as the mean increases or decreases (refer to Reed et al. 2002). Therefore, acceptance sampling plans considering the CV as the reliability parameter can complement each other with the other acceptance sampling plans, so as to control the product quality and improve the management level.

CV can be applied not only characteristic analysis of ultimate strength or fatigue limit, failure rates and structural/material reliability, but also for both the reliability-based design of mechanical systems or components and the evaluation of an existing product (see for He and Oyadiji 2001). In the fields of materials engineering and manufacturing, Castagliola et al. (2015) have stated that some quality characteristics related to the physical properties of products often have a standard deviation that is proportional to their population mean. Tool cutting life and several properties of sintered materials are some typical examples. In such scenarios, the CV remains constant even though the mean and standard deviation may change from one sample to another. Zhang (1989) pointed that the CV can be predetermined from the long term of engineering practice in the research of structural reliability design, evaluation, and inspection.

CV is a good measure of the reliability of experiments, that is, the smaller the CV value, the higher the reliability (Steel and Torrie 1980; Taye and Njuho 2008). Recently, Ma and Zhang (1997) deduced the CV method for structural reliability inspection using the CV as the quality control parameter, under the condition of the CV being known. The inspection efficiency of CV method is higher than S method and $\sigma$ method. Tong and Chen (1991) proposed a variable single sampling plan using CV to evaluate the quality stability of normally distributed products. Yan et al. (2016) developed a variable two stage sampling plan based on CV, which is more efficient than the single sampling plan proposed by Tong and Chen (1991).

In advanced manufacturing processes, supplier production is frequently continuous, so the quality of preceding and/or successive lots is expected to be homogeneous and dependent (Kuraimani and Govindaraju 1992). But the single sampling plan and the two stage sampling plan only consider the present state of a lot, that is, they accept or reject a lot based on the present lot quality. In order to compensate for this weaknesses, Wortham and Baker (1976) introduced the multiple dependent state (MDS) sampling plan, which examines a lot based on not only the sample information from the current lot but also the quality of preceding lots. So the MDS sampling plan can be used in the case that lots are submitted for inspection serially. Recently, Balamurali and Jun (2007) proposed MDS sampling plan by variables for the assessment of normally distributed quality characteristics. Aslam et al. (2015) proposed a mixed MDS sampling plan using the process capability index, and Aslam et al. (2014) considered MDS sampling for the development of a new attribute control chart. To the best of our knowledge, there exist no studies about the MDS plan based on the CV. Therefore, assuming that the quality characteristic follows the normal distribution, we will develop the MDS sampling plan using the CV with expectation that it is more efficient than the single plan proposed by Tong and Chen (1991) in this article.

## Multiple dependent state (MDS) sampling plan

The coefficient of variation (CV) is a statistic defined as the ratio of the standard deviation $\sigma$ to the mean $\mu$. Suppose that the quality of interest *X* follows a normal distribution with the mean of $\mu$ and the variance of $\sigma^{2}$, the CV of the random variable *X* is defined as

1 $$\gamma = {\sigma \mathord{\left/ {\vphantom {\sigma \mu }} \right. \kern-0pt} \mu }$$

Assume that $X_{1} ,X_{2} , \ldots ,X_{n}$ is a sample of the normal distribution $N(\mu ,\sigma^{2} )$, then the sample coefficient of variation is defined as

2 $$\hat{\gamma } = \frac{S}{{\bar{X}}}$$

where $S = \sqrt {\frac{1}{n - 1}\sum\nolimits_{i = 1}^{n} {(X_{i} - \bar{X})^{2} } }$ is the sample standard deviation, $\bar{X} = \sum\nolimits_{i = 1}^{n} {X_{i} /n}$ is the sample mean.

Iglewicz et al. (1968) noticed that the statistic $\sqrt n /\hat{\gamma }$ follows the noncentral *t* distribution, i.e. $\sqrt n /\hat{\gamma }\sim t(n - 1,\sqrt n /\gamma )$, where *n* − 1 is the degrees of freedom, and $\sqrt n /\gamma$ is the noncentrality parameter. Denote the cumulative distribution function (cdf) of $\hat{\gamma }$ as

3 $$F_{{\hat{\gamma }}} (u\left| {n,\gamma } \right.) = 1 - F_{t} \left( {\frac{\sqrt n }{u}\left| {n - 1,\frac{\sqrt n }{\gamma }} \right.} \right)$$

where $F_{t} ( \cdot )$ is the cdf of the $t(n - 1,\sqrt n /\gamma )$ distribution.

Steel and Torrie (1980), Taye and Njuho (2008) point that the CV is a good measure of the reliability of the experiment. Here we use the CV as the quality benchmark for acceptance of a product lot. Let $v_{1}$ and $v_{2}$ denote the quality level of AQL (acceptable quality level) and LQL (limiting quality level) based on the CV, respectively. Then the operating procedure of the proposed plan based on the CV is stated as follows:

- **Step 1**: Choose the values of $(v_{1} ,v_{2} )$ based on the CV at producer’s risk $\alpha$ and consumer’s risk $\beta$.
- **Step 2:** Select a random sample of size *n*, ($X_{1} ,X_{2} , \ldots ,X_{n}$), from the lot, then compute the sample CV $\hat{\gamma }$ defined in (2).
- **Step 3**: Accept the entire lot if $\hat{\gamma } \le k_{a}$, reject the lot if $\hat{\gamma } > k_{r}$; if $k_{a} < \hat{\gamma } \le k_{r}$, then accept the current lot provided that the proceeding *m* lots have been accepted under the condition of $\hat{\gamma } \le k_{a}$, otherwise reject the lot. Note that $k_{a}$ and $k_{r}$ are acceptance constant and rejection constant, respectively.

The proposed plan is characterized by four parameters $k_{a}$, $k_{r}$, *m* and *n*. If $k_{a} = k_{r}$, then it reduces to an ordinary variable single sampling plan proposed by Tong and Chen (1991) .

According to Balamurali and Jun (2007), the OC function of the proposed MDS sampling plan is

4 $$P_{a} (v) = P\left\{ {\hat{\gamma } \le k_{a} \left| {\gamma = v} \right.} \right\} + P\{ k_{a} < \hat{\gamma } \le k_{r} \left| {\gamma = v} \right.\} [P\{ \hat{\gamma } \le k_{a} \left| {\gamma = v} \right.\} ]^{m}$$

The lot acceptance probability using single sampling and the probability of rejecting the lot directly based on the CV are respectively given as follows

$$P\left\{ {\hat{\gamma } \le k_{a} \left| {\gamma = v} \right.} \right\} = 1 - F_{t} \left( {\frac{\sqrt n }{{k_{a} }}\left| {n - 1,\frac{\sqrt n }{v}} \right.} \right)$$

$$P\{ \hat{\gamma } > k_{r} \left| {\gamma = v} \right.\} = F_{t} \left( {\frac{\sqrt n }{{k_{r} }}\left| {n - 1,\frac{\sqrt n }{v}} \right.} \right)$$

So,

$$P\{ k_{a} < \hat{\gamma } \le k_{r} \left| {\gamma = v} \right.\} = P\{ \hat{\gamma } \le k_{r} \left| {\gamma = v} \right.\} - P\{ \hat{\gamma } \le k_{a} \left| {\gamma = v} \right.\} = F_{t} \left( {\frac{\sqrt n }{{k_{a} }}\left| {n - 1,\frac{\sqrt n }{v}} \right.} \right) - F_{t} \left( {\frac{\sqrt n }{{k_{r} }}\left| {n - 1,\frac{\sqrt n }{v}} \right.} \right)$$

Then the OC function of the MDS sampling plan can be rewritten as

5 $$P_{a} (v) = 1 - F_{t} \left( {\frac{\sqrt n }{{k_{a} }}\left| {n - 1,\frac{\sqrt n }{v}} \right.} \right) + \left[ {F_{t} \left( {\frac{\sqrt n }{{k_{a} }}\left| {n - 1,\frac{\sqrt n }{v}} \right.} \right) - F_{t} \left( {\frac{\sqrt n }{{k_{r} }}\left| {n - 1,\frac{\sqrt n }{v}} \right.} \right)} \right]\left[ {1 - F_{t} \left( {\frac{\sqrt n }{{k_{a} }}\left| {n - 1,\frac{\sqrt n }{v}} \right.} \right)} \right]^{m}$$

## Determination of the proposed sampling plan parameters

Yen and Chang (2009) stated “A well-designed sampling plan must provide a probability of at least ($1 - \alpha$) of accepting a lot if the product quality level is $v_{1}$ and a probability of no more than $\beta$ of accepting a lot if the level of the product quality is $v_{2}$.” Thus, the OC curve of the proposed variables MDS plan will be designed to pass through two designated points, ($v_{1}$, $1 - \alpha$) and ($v_{2}$, $\beta$). For the specified $\alpha$, $\beta$, $v_{1}$ and $v_{2}$, the proposed MDS sampling plan parameters must satisfy the following two inequalities

6 $$P_{a} (v_{1} ) = \, \Pr \{ {\text{Accepting}}\,{\text{the}}\,{\text{lot}}\left| {\gamma = v_{1} } \right.\} \ge 1 - \alpha$$

7 $$P_{a} (v_{2} ) = \Pr \{ {\text{Accepting}}\,{\text{the}}\,{\text{lot}}\left| {\gamma = v_{2} } \right.\} \le \beta$$

Since there are several combinations of the parameters for the proposed plans which satisfy the above two inequations, we choose the designed parameters which minimize the sample size. The parameters $k_{a}$, $k_{r}$ and *n* of the proposed plan can be obtained by solving the following optimization problem:

8 $$\begin{aligned}& \quad Minimize \quad n \\ & {\text{s.t}} \\ & \left\{ {\begin{array}{l} 1 - F_{t} \left( {\frac{\sqrt n }{{k_{a}}}\left| {n - 1,\frac{\sqrt n }{{v_{1} }}} \right.} \right) + \left[{F_{t} \left( {\frac{\sqrt n }{{k_{a} }}\left| {n - 1,\frac{\sqrt n}{{v_{1} }}} \right.} \right) - F_{t} \left({\frac{\sqrt n }{{k_{a}}}\left| {n - 1,\frac{\sqrt n }{{v_{1} }}}\right.} \right)}\right]\left[ {1 - F\left( {\frac{\sqrt n }{{k_{a}}}\left| {n -1,\frac{\sqrt n }{{v_{1} }}} \right.} \right)_{t} }\right]^{m} \ge1 - \alpha \\ 1 - F_{t} \left( {\frac{\sqrt n}{{k_{a} }}\left|{n - 1,\frac{\sqrt n }{{v_{2} }}} \right.} \right) + \left[ {F_{t}\left( {\frac{\sqrt n }{{k_{a} }}\left| {n -1,\frac{\sqrt n}{{v_{2} }}} \right.} \right) - F_{t} \left({\frac{\sqrt n }{{k_{r}}}\left| {n - 1,\frac{\sqrt n }{{v_{2} }}}\right.} \right)}\right]\left[ {1 - F_{t} \left( {\frac{\sqrt n}{{k_{a} }}\left| {n- 1,\frac{\sqrt n }{{v_{2} }}} \right.} \right)}\right]^{m} \le\beta \\ n \ge 2,v_{1} < v_{2} ,0 \le k_{a} \le k_{r} \\ \end{array} } \right.\end{aligned}$$

**Fig. 1:**
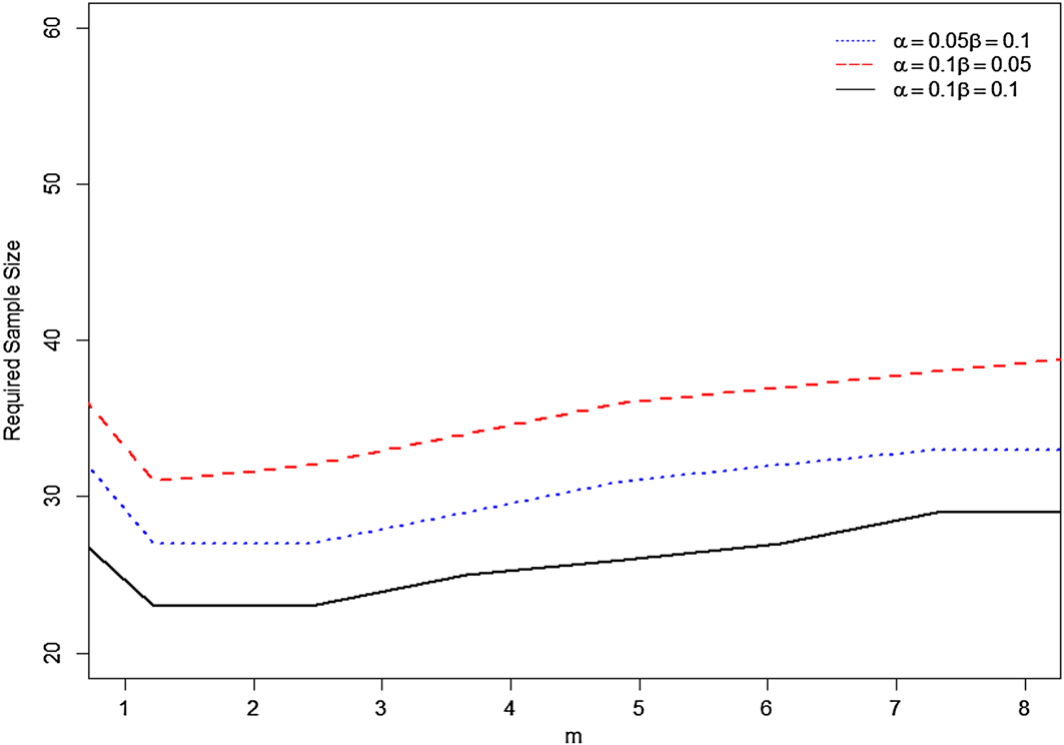
Required sample size *n* of MDS sampling plan with *m* = 1–8

In order to investigate the effect of different *m* values on the required sample size of the proposed MDS sampling plan, we vary *m* from 1 to 8. Figure 1 shows the required sample size *n* varies with the *m* value under $(v_{1} ,v_{2} )$ = (0.05, 0.07), $(\alpha ,\beta )$ = (0.05, 0.10), (0.10, 0.05) and (0.10, 0.10). From Fig. 1, we see that the required sample size *n* decreases with the increase of $\beta$ value (or $\alpha$ value) for fixed the value of $\alpha$(or $\beta$). That is to say, the larger the risk tolerance, the smaller the sample size required to ensure the same quality level. In addition, the required sample sizes do not change much under the different *m* values for each set of risk values.

 Referring to the values of CV selected by Kang et al. (2007) and Tong and Chen (1991), we consider $v_{1}$ = 0.05, 0.06, 0.07, 0.08, 0.09, 0.10, $v_{2}$ = 0.06 ~ 0.12 here. The proposed sampling plan parameters (*n*, $k_{a}$, $k_{r}$) with schemes *m* = 1, 2, 3 are respectively displayed in Tables 1, 2 and 3 for $(\alpha,\,\beta )$ = (0.05, 0.10), (0.10, 0.05) and (0.10, 0.10). From the results of Tables 1, 2 and 3, we note that the corresponding sample size *n* decreases when $v_{2}$ value increases for fixed values of $\alpha$, $\beta$ and $v_{1}$. On the other hand, for fixed $\alpha$, $\beta$ and $v_{2}$, the corresponding sample size *n* increases when $v_{1}$ value increases. For example, when *m* = 3, $v_{1}$ = 0.06, ($\alpha$, $\beta$) = (0.05, 0.10), *n* = 127 as $v_{2}$ = 0.07, and for all other same values, *n* = 8 when $v_{2}$ = 0.12. On the other hand, when *m* = 3, $v_{2}$ = 0.08, ($\alpha$, $\beta$) = (0.05, 0.10), *n* = 16 as $v_{1}$ = 0.05, and for all other same values, *n* = 167 when $v_{1}$ = 0.07.

**Table 1:** The proposed plan parameters under (*α*, *β*) = (0.05, 0.10), (0.10, 0.05), (0.10, 0.10) (*m* = 1)

*v*_1_	*v*_2_	(*α*, *β*)								
		(0.05, 0.10)			(0.10, 0.10)			(0.10, 0.05)		
		*k*_*a*_	*k*_*r*_	n	*k*_*a*_	*k*_*r*_	n	*k*_*a*_	*k*_*r*_	n
0.05	0.06	0.05318	0.05849	85	0.05166	0.06078	95	0.05216	0.05888	70
	0.07	0.05561	0.06530	28	0.05342	0.06279	30	0.05336	0.06758	22
	0.08	0.05693	0.07151	15	0.05412	0.06777	17	0.0557	0.0667	13
	0.09	0.05771	0.08538	10	0.05466	0.07324	12	0.05759	0.06885	9
	0.10	0.05867	0.09885	8	0.05475	0.09235	9	0.05665	0.08375	7
	0.11	0.05970	0.10620	7	0.05611	0.09931	8	0.05527	0.09801	6
	0.12	0.06081	0.12030	6	0.05821	0.10770	7	0.05941	0.07568	5
0.06	0.07	0.06314	0.06938	120	0.06181	0.06947	132	0.06247	0.06793	95
	0.08	0.06516	0.07975	36	0.06311	0.07612	40	0.06381	0.07678	29
	0.09	0.06732	0.08352	19	0.06470	0.07958	22	0.06485	0.08342	16
	0.10	0.06850	0.09399	13	0.06588	0.08128	14	0.06787	0.08078	11
	0.11	0.06975	0.10900	10	0.06528	0.10460	11	0.0661	0.1106	9
	0.12	0.07055	0.11010	8	0.06558	0.10500	9	0.06854	0.1005	7
0.07	0.08	0.07302	0.08102	159	0.07232	0.07648	172	0.07233	0.07918	128
	0.09	0.07551	0.08876	46	0.07370	0.08416	52	0.07395	0.0871	38
	0.10	0.07792	0.09311	25	0.07471	0.08945	27	0.07599	0.09193	21
	0.11	0.07916	0.10670	16	0.07535	0.09831	17	0.07595	0.1029	13
	0.12	0.08030	0.11050	12	0.07572	0.11050	13	0.07692	0.1063	10
0.08	0.09	0.08329	0.08939	203	0.08204	0.08777	218	0.08249	0.0885	161
	0.10	0.08558	0.09992	60	0.08342	0.09748	64	0.08397	0.1000	48
	0.11	0.08772	0.10650	30	0.08491	0.10060	33	0.08519	0.1085	25
	0.12	0.09045	0.10820	19	0.08521	0.11070	22	0.08751	0.1056	16
0.09	0.10	0.09318	0.09987	255	0.09218	0.09861	273	0.09244	0.09902	197
	0.11	0.09646	0.10590	73	0.09346	0.10820	78	0.09414	0.1087	57
	0.12	0.09829	0.11530	37	0.09476	0.11360	40	0.09587	0.1149	30
0.10	0.11	0.10320	0.10990	307	0.10210	0.10840	330	0.1023	0.1103	240
	0.12	0.10580	0.12160	89	0.10380	0.11560	94	0.1046	0.1162	69

**Table 2:** The proposed plan parameters under (*α*, *β*) = (0.05, 0.10), (0.10, 0.05), (0.10, 0.10) (*m* = 2)

*v*_1_	*v*_2_	(*α*, *β*)								
		(0.05, 0.10)			(0.10, 0.10)			(0.10, 0.05)		
		*k*_*a*_	*k*_*r*_	*n*	*k*_*a*_	*k*_*r*_	*n*	*k*_*a*_	*k*_*r*_	*n*
0.05	0.06	0.05361	0.06971	87	0.05257	0.06966	97	0.05298	0.08101	72
	0.07	0.05656	0.10760	28	0.05437	0.06490	32	0.05524	0.07025	23
	0.08	0.05925	0.09898	16	0.05540	0.08372	18	0.05681	0.08024	13
	0.09	0.06141	0.10630	11	0.05707	0.08895	13	0.05754	0.08661	9
	0.10	0.06247	0.09962	8	0.05765	0.10510	9	0.06024	0.07335	7
	0.11	0.06400	0.11040	7	0.05776	0.11090	8	0.05898	0.1057	6
	0.12	0.06360	0.11140	6	0.06097	0.11960	7	0.05969	0.1041	5
0.06	0.07	0.06372	0.07712	121	0.06269	0.07227	134	0.06313	0.07553	98
	0.08	0.06673	0.08421	39	0.06455	0.08179	41	0.06534	0.1322	31
	0.09	0.06893	0.09754	20	0.06634	0.09493	23	0.06716	0.1025	17
	0.10	0.07102	0.09844	13	0.06739	0.10130	16	0.06908	0.09683	11
	0.11	0.07373	0.10640	10	0.06842	0.11550	11	0.07035	0.1206	9
	0.12	0.07435	0.11480	8	0.06888	0.12110	9	0.07174	0.1052	7
0.07	0.08	0.07374	0.08281	162	0.07260	0.08323	175	0.07309	0.1294	129
	0.09	0.07715	0.08884	48	0.07481	0.09545	53	0.07545	0.1254	40
	0.10	0.07950	0.10380	25	0.07640	0.09878	29	0.07711	0.1305	22
	0.11	0.08203	0.12330	17	0.07739	0.11040	19	0.07909	0.1011	14
	0.12	0.08407	0.12380	12	0.07914	0.12240	14	0.07968	0.1198	11
0.08	0.09	0.08393	0.09348	206	0.08274	0.09897	227	0.08312	0.1015	161
	0.10	0.08725	0.10110	61	0.08477	0.10510	67	0.08584	0.1188	49
	0.11	0.08962	0.12350	31	0.08678	0.11840	35	0.08735	0.114	26
	0.12	0.09241	0.12200	20	0.08791	0.12830	23	0.08927	0.1231	17
0.09	0.10	0.09382	0.14880	257	0.09275	0.10150	277	0.09314	0.1126	201
	0.11	0.09735	0.11020	74	0.09501	0.11530	81	0.09572	0.12	58
	0.12	0.10060	0.11770	38	0.09698	0.12370	42	0.09813	0.1413	32
0.10	0.11	0.10400	0.11200	312	0.10270	0.11970	334	0.1032	0.1441	246
	0.12	0.10760	0.1286	90	0.10510	0.12140	97	0.1061	0.121	72

**Table 3:** The proposed plan parameters under (*α*, *β*) = (0.05, 0.10), (0.10, 0.05), (0.10, 0.10) (*m* = 3)

*v*_1_	*v*_2_	(*α*, *β*)								
		(0.05, 0.10)			(0.10, 0.10)			(0.10, 0.05)		
		*k*_*a*_	*k*_*r*_	*n*	*k*_*a*_	*k*_*r*_	*n*	*k*_*a*_	*k*_*r*_	*n*
0.05	0.06	0.05395	0.06719	92	0.05288	0.06051	103	0.05339	0.05934	74
	0.07	0.05710	0.06966	29	0.05489	0.0814	33	0.05566	0.0761	24
	0.08	0.05949	0.08085	16	0.05670	0.08502	19	0.05782	0.0805	14
	0.09	0.06236	0.09867	11	0.05753	0.09749	13	0.05911	0.07918	9
	0.10	0.06327	0.09177	8	0.05951	0.10750	10	0.06029	0.1294	7
	0.11	0.06551	0.11210	7	0.05955	0.11660	8	0.06088	0.1195	6
	0.12	0.06732	0.12820	6	0.06107	0.12420	7	0.06602	0.08333	6
0.06	0.07	0.06414	0.07668	127	0.06297	0.07011	142	0.06342	0.1292	101
	0.08	0.06735	0.08271	39	0.06518	0.08149	44	0.06608	0.09012	32
	0.09	0.06995	0.09504	21	0.06706	0.09177	24	0.06793	0.1465	18
	0.10	0.07306	0.09381	14	0.06839	0.09786	17	0.06969	0.09208	12
	0.11	0.07423	0.11220	10	0.06969	0.10520	12	0.0723	0.1028	9
	0.12	0.07568	0.12390	8	0.07129	0.12060	10	0.07275	0.09233	8
0.07	0.08	0.07419	0.08536	167	0.07303	0.08775	189	0.07346	0.09107	135
	0.09	0.07792	0.08845	52	0.07556	0.09699	58	0.07637	0.1125	41
	0.10	0.08034	0.09316	27	0.07740	0.10450	31	0.07831	0.1091	22
	0.11	0.08359	0.11960	17	0.07901	0.11100	20	0.08043	0.09617	15
	0.12	0.08486	0.11550	12	0.08128	0.12160	15	0.08309	0.1181	11
0.08	0.09	0.08421	0.10350	217	0.08304	0.09478	240	0.08354	0.09341	171
	0.10	0.08770	0.10430	63	0.08551	0.10920	70	0.08666	0.1078	52
	0.11	0.09088	0.11450	32	0.08806	0.11210	38	0.08893	0.1376	27
	0.12	0.09319	0.12680	20	0.08932	0.12470	24	0.09183	0.1431	18
0.09	0.10	0.09426	0.10830	266	0.09307	0.11120	299	0.09357	0.1264	214
	0.11	0.09768	0.11610	78	0.09557	0.11660	86	0.09656	0.1206	62
	0.12	0.10130	0.12490	39	0.09777	0.12680	45	0.09903	0.1442	32
0.10	0.11	0.10430	0.11740	327	0.10310	0.11850	366	0.1036	0.1188	262
	0.12	0.10800	0.12940	92	0.10570	0.12870	105	0.1066	0.1477	74

## Advantages of the MDS plan

In this section, we will use these two criteria, the OC curves and the sample size required for inspection, to demonstrate the advantages of the proposed MDS plan over the single plan proposed by Tong and Chen (1991).

### OC curves

**Fig. 2:**
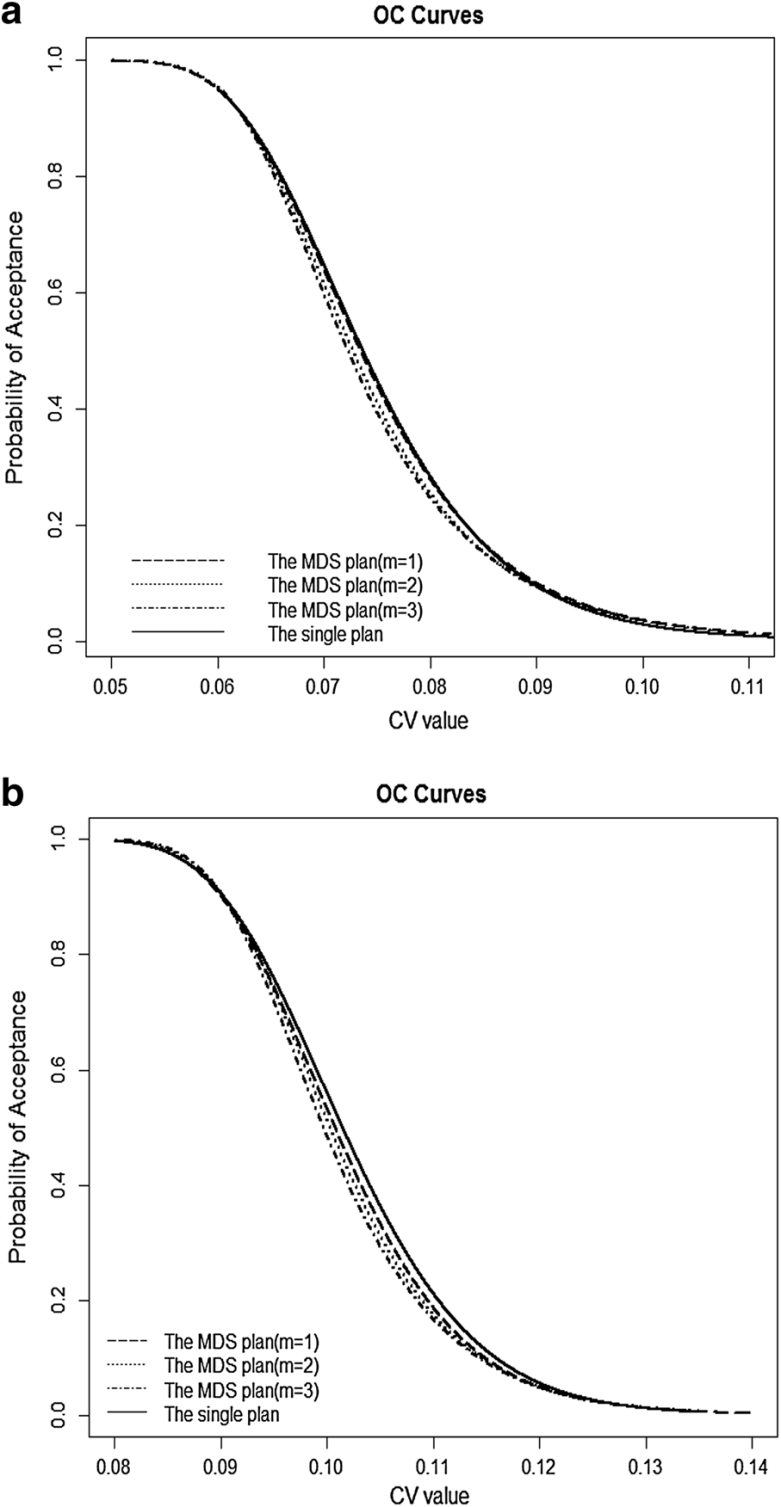
OC curves of MDS plan (*m* = 1, 2, 3) and single plan for different quality and risk parameters: **a** ($v_{1}$, $v_{2}$) = (0.06, 0.09), ($\alpha$, $\beta$) = (0.05,0.10). **b** ($v_{1}$, $v_{2}$) = (0.09, 0.12), ($\alpha$, $\beta$) = (0.10,0.05)

In order to show the efficiency of the proposed sampling plan, Fig. 2 displays the OC curves of the MDS plan (*m* = 1, 2, 3) and the single sampling plan for two cases: (a) ($v_{1}$, $v_{2}$) = (0.06, 0.09), ($\alpha$, $\beta$) = (0.05, 0.10), (b) ($v_{1}$, $v_{2}$) = (0.09, 0.12), ($\alpha$, $\beta$) = (0.10, 0.05). In Fig. 2, we can see that the four curves of the sampling plans are very similar in case (a) or in case (b), but the sample size required by the MDS sampling plan is much fewer. For example, the single plan requires *n* = 28 while the MDS plan with *m* = 1 requires *n* = 19 in case (a). In addition, all of the OC curves show that the probability of acceptance will become smaller as the value of CV increases, which is as expected from the theory. Since the MDS sampling plan requires fewer sample size to give the desired protection, the cost of inspection will greatly be reduced. Therefore, it is reasonable to conclude the MDS plan has a better performance.

### Sample sizes required for inspection

**Fig. 3:**
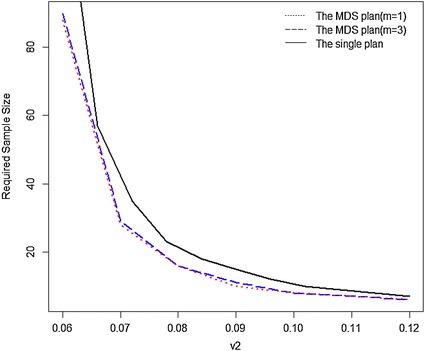
Required sample sizes of MDS plan (*m* = 1, 3) and single plan for $\alpha$ = 0.05, $\beta$ = 0.10, $v_{1}$ = 0.05

**Fig. 4:**
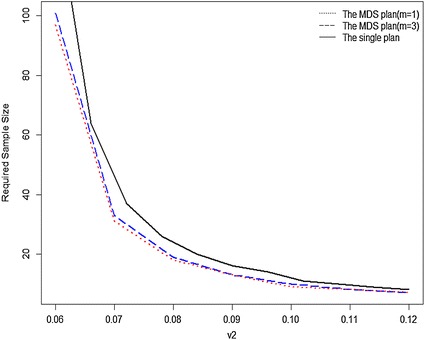
Required sample sizes of MDS plan (*m* = 1, 3) and Single plan for $\alpha$ = 0.10, $\beta$ = 0.05, $v_{1}$ = 0.05

In order to compare the sample sizes required for inspection in the MDS plan (*m* = 1, 3) and the single plan with different values of $v_{1}$ and $v_{2}$, the $v_{1}$ value is fixed at 0.05 and $v_{2}$ value increases from 0.06 to 0.12. The results are showed in Fig. 3 ($\alpha$ = 0.05, $\beta$ = 0.10) and Fig. 4 ($\alpha$ = 0.10, $\beta$ = 0.05). From Figs. 3 and 4, the required sample size *n* of three sampling plans all decreases as the value of $v_{2}$ rises from 0.06 to 0.12. Clearly, the required sample size *n* is larger as the value of $v_{2}$ is closer to the value of $v_{1}$. Moreover, we also find that the single sampling plan requires more samples than the MDS plans when $v_{2}$ takes any value between 0.06 and 0.12. Therefore, the MDS sampling plan is a more cost-effective plan while the single plan is relatively uneconomical.

On the other side, we also list the sample sizes required for the single sampling plan and MDS plan (*m* = 1, 2, and 3) in Table 4 with commonly used values of $v_{1}$ and $v_{2}$ when $(\alpha ,\beta )$ = (0.05, 0.10), (0.10, 0.05) and (0.10, 0.10). From Table 4, it is obvious that the sample size required by the MDS plan is fewer than required by the single sampling plan for all cases. For example, when $v_{1}$ = 0.08, $v_{2}$ = 0.09, $(\alpha ,\beta )$ = (0.10, 0.05), the sample size of the MDS plan is 218 for *m* = 1, 227 for *m* = 2, and 240 for *m* = 3, while the single plan is 318. Therefore, the proposed sampling plan will give the desired protection with the less required sample size so that the MDS plan is economically superior to the single plan.

**Table 4:** The comparison of sample size of two sampling plans with (*α*, *β*) = (0.05, 0.10), (0.10, 0.05), (0.10, 0.10)

*v*_1_	*v*_2_	$\alpha = 0. 0 5$, $\beta = 0.10$				$\alpha = 0. 1 0$, $\beta = 0. 0 5$				$\alpha = 0. 1 0$, $\beta = 0. 1 0$			
		*m* = 1	*m* = 2	*m* = 3	*n*	*m* = 1	*m* = 2	*m* = 3	*n*	*m* = 1	*m* = 2	*m* = 3	*n*
0.05	0.06	85	87	92	131	95	97	103	134	70	72	74	101
	0.07	28	28	29	39	30	32	33	41	22	23	24	31
	0.08	15	16	16	20	17	18	19	23	13	13	14	17
	0.09	10	11	11	14	12	13	14	15	9	9	9	12
	0.10	8	8	8	11	9	9	10	12	7	7	7	9
	0.11	7	7	7	9	8	8	8	9	6	6	6	8
	0.12	6	6	6	7	7	7	7	8	5	5	6	7
0.06	0.07	120	121	127	182	132	134	142	186	95	98	101	142
	0.08	36	39	39	53	40	41	44	56	29	31	32	42
	0.09	19	20	21	28	22	23	24	28	16	17	18	22
	0.10	13	13	14	17	14	16	17	20	11	11	12	15
	0.11	10	10	10	14	11	11	12	15	9	9	9	11
	0.12	8	8	8	11	9	9	10	12	7	7	8	9
0.07	0.08	159	162	167	242	172	175	189	248	128	129	135	188
	0.09	46	48	52	69	52	53	58	72	38	40	41	55
	0.10	25	25	27	35	27	29	31	36	21	22	22	28
	0.11	16	17	17	23	17	19	20	25	13	14	15	19
	0.12	12	12	12	17	13	14	15	17	10	11	11	14
0.08	0.09	203	206	217	311	218	227	240	318	161	161	171	242
	0.10	60	61	63	88	64	67	70	91	48	49	52	69
	0.11	30	31	32	44	33	35	38	46	25	26	27	35
	0.12	19	20	20	28	22	23	24	30	16	17	18	23
0.09	0.10	255	257	266	327	273	277	299	336	197	201	214	303
	0.11	73	74	78	109	78	81	86	112	57	58	62	85
	0.12	37	38	39	55	40	42	45	56	30	32	32	44
0.10	0.11	307	312	327	335	330	334	366	343	240	246	262	335
	0.12	89	90	92	132	94	97	105	136	69	72	74	103

## An illustrative example

To illustrate the proposed MDS plan for practical applications, we use the actual data as discussed by Aslam et al. (2013). The data is about concrete which is widely used to construct buildings, roads, and a variety of other structures. The compressive strength of concrete is the most common quality measure used by the engineer in designing buildings and other structures. In the contract formulated from the producer and the consumer, suppose that the producer requires the probability of accepting the concrete at least 95 % if the CV of the compressive strength is less than 0.08, and the consumer require that the probability of accepting the concrete would be no more than 10 % if the CV of the compressive strength is larger than 0.12. That is, the values of $v_{1}$ and $v_{2}$ are set to 0.08 and 0.12 with the producer’s risk $\alpha$ = 0.05 and the consumer’s risk $\beta$ = 0.10. Therefore, the problem is the determination of the acceptance constants and the inspected sample sizes that provide the desired levels of protection for both producers and consumers.

Based on our proposed methodology, we can obtain the plan parameters as (*n*, $k_{a}$, $k_{r}$) = (20, 0.09241, 0.122) from Table 2 considering the MDS plan with *m* = 2. Hence, the 20 inspected samples are taken from the lot randomly and the compressive strength of these 20 concrete mixture specimens is measured and displayed in Table 5. Aslam et al. (2013) have showed that these observed measurements are fairly close to the normal distribution. Based on the collected 20 measurements, we have

$$\overline{X} = 32.19,\quad S = 3.843,\quad {\text{and}}\quad \hat{\gamma } = S/\overline{X} = 0.1194.$$

Since $k_{a} < \hat{\gamma } = 0.1194 < k_{r}$, the consumer will accept the lot provided that the proceeding *m* (= 2) lots have been accepted under the condition of $\hat{\gamma } \le k_{a}$, otherwise, reject the lot. Moreover, we note that if the single sampling plan (Tong and Chen 1991) based on the CV are applied to this case, the sample size required for inspection is 28 under the same conditions.

**Table 5:** The compressive strength of 20 concrete mixture specimens

36.3	40.1	31.8	33.6	34.9	31.2	32.8	25.8	30.8	32.9
30.9	31.9	35.6	30.9	27.8	24.9	31.6	27.9	33.7	38.4

## Conclusions

In this paper, a multiple dependent state (MDS) sampling plan for accepting a lot whose quality characteristic follows a normal distribution based on the coefficient of variation (CV) is presented. Several tables are given for practical use. By comparison with the single sampling plan propose by Tong and Chen (1991) in terms of the required sample size and the OC curve, which show that our proposed MDS plan has a better performance than the single plan. Hence, the industrialists can save the inspection cost if they use the proposed MDS plan. Finally, a real example shows the application of the proposed plan in various industries. The present study can be extended for non-normal distribution as future research.
